# A hybrid approach for predicting transcription factors

**DOI:** 10.3389/fbinf.2024.1425419

**Published:** 2024-07-25

**Authors:** Sumeet Patiyal, Palak Tiwari, Mohit Ghai, Aman Dhapola, Anjali Dhall, Gajendra P. S. Raghava

**Affiliations:** Department of Computational Biology, Indraprastha Institute of Information Technology, New Delhi, India

**Keywords:** transcription factor, alignment-free methods, alignment-based methods, regulation of transcription, hybrid method, DNA-binding proteins

## Abstract

Transcription factors are essential DNA-binding proteins that regulate the transcription rate of several genes and control the expression of genes inside a cell. The prediction of transcription factors with high precision is important for understanding biological processes such as cell differentiation, intracellular signaling, and cell-cycle control. In this study, we developed a hybrid method that combines alignment-based and alignment-free methods for predicting transcription factors with higher accuracy. All models have been trained, tested, and evaluated on a large dataset that contains 19,406 transcription factors and 523,560 non-transcription factor protein sequences. To avoid biases in evaluation, the datasets were divided into training and validation/independent datasets, where 80% of the data was used for training, and the remaining 20% was used for external validation. In the case of alignment-free methods, models were developed using machine learning techniques and the composition-based features of a protein. Our best alignment-free model obtained an AUC of 0.97 on an independent dataset. In the case of the alignment-based method, we used BLAST at different cut-offs to predict the transcription factors. Although the alignment-based method demonstrated excellent performance, it was unable to cover all transcription factors due to instances of no hits. To combine the strengths of both methods, we developed a hybrid method that combines alignment-free and alignment-based methods. In the hybrid method, we added the scores of the alignment-free and alignment-based methods and achieved a maximum AUC of 0.99 on the independent dataset. The method proposed in this study performs better than existing methods. We incorporated the best models in the webserver/Python Package Index/standalone package of “TransFacPred” (https://webs.iiitd.edu.in/raghava/transfacpred).

## 1 Introduction

Transcription factors (TFs) are DNA-binding proteins that bind to specific DNA segments to control the expression of the genes (Ortet et al., 2012; Lambert et al., 2018; Miyazaki and Miyazaki, 2021). These TFs or regulators control specific cell types, cell differentiation, gene regulatory pathways, and immune responses (Fong and Tapscott, 2013; Lee and Young, 2013; Singh et al., 2014). Recognition of TFs is the first step in understanding the transcription regulatory system (Kim et al., 2021). Mis-regulation and mutations in TFs or their binding regions lead to the development of disorders like Rubinstein–Taybi, CHOPS syndromes, Coffin–Siris, etc. (Lee and Young, 2013; Sim et al., 2015; Izumi, 2016; Kircher et al., 2019). Several biological mechanisms such as chromosomal translocation, aberrant gene expression, point substitutions, and mutations associated with the non-coding DNA result in the alteration of transcription factor binding sites in various cancer types (Kleinjan and van Heyningen, 2005; Herceg and Hainaut, 2007; Bushweller, 2019; Jiramongkol and Lam, 2020; Kishtagari et al., 2020). In addition, several inflammatory autoimmune diseases and improper immune development are associated with the misregulation of the NF-kB transcription factor (Hayden and Ghosh, 2012). Studies have also revealed that, with a better understanding of the transcriptional regulations, it is possible to control gene expression in various genetic perturbations (Munsky et al., 2012; Lee and Young, 2013; Kemmeren et al., 2014). Several attempts in clinical research have been made to target, inhibit, or modulate transcription factor DNA-binding activity in various disease conditions (Bhagwat and Vakoc, 2015; Cheng et al., 2019; Li et al., 2020).

With the availability of enormous genome sequencing datasets, many methods have been developed to identify TFs (Pereira et al., 2020). It is not feasible to identify TFs in genomics using experimental techniques. In order to overcome these limitations, a number of *in silico* methods have been developed to annotate TFs at the genome scale (Odom, 2011). Zheng and colleagues developed a hybrid strategy utilizing support vector machine (SVM) and error-correcting output coding (ECOC) algorithms to predict distinct categories of TFs, such as helix-turn-helix, beta-scaffold, and zinc-coordinating DNA-binding domains (Zheng et al., 2008). Eichner and colleagues developed a four-step workflow that implemented two complementary tools, TFpredict and SABINE, for identifying the DNA-binding domains and discovering the DNA motif in a protein. TFpredict uses machine/deep learning techniques to predict a transcription factor (Eichner et al., 2013). Another tool, BART, has been developed to predict functional factors that bind at cis-regulatory regions from a gene list or a ChIP-seq dataset (Wang et al., 2018). Recently, Kim et al. developed DeepTFactor, a deep learning-based tool that predicts TFs using a convolutional neural network (Kim et al., 2021). That study created and used the largest possible dataset to develop an accurate and reliable method. The existing methods are computationally expensive and need domain expertise (e.g., understanding sources, types of information, and limitations of the data).

In order to overcome the limitations of existing methods, we developed an improved method for predicting transcription factors with high accuracy. Initially, we developed homology or alignment-based methods for the prediction of the TFs. These alignment-based methods exhibit high performance if the query TF has high similarity with the target TFs in the database. However, these methods fail if a query TF has either poor similarity with the known TFs in the database or high similarity with non-TFs. We developed an alignment-free method to overcome these limitations. In alignment-free methods, different machine learning techniques are used to build prediction models using the composition of TFs as an input feature. To combine the power of both alignment-free and alignment-based methods, we developed a hybrid method. The hybrid method leverages the efficiency and scalability of alignment-free techniques while incorporating the precision of alignment-based approaches, aiming to maximize predictive performance and overcome the limitations inherent in using either method alone. This integrated strategy ensures robust and comprehensive analysis, enhancing the accuracy and reliability of transcription factor predictions. To support the scientific community, we developed the web server and standalone software package TransFacPred, which is freely available at https://webs.iiitd.edu.in/raghava/transfacpred and https://github.com/raghavagps/transfacpred for predicting transcription factors from protein sequences.

## 2 Materials and methods

### 2.1 Dataset collection and preprocessing

We obtained the TF and non-TF protein sequence dataset, which was released in September 2019, from the UniProt Knowledgebase (UniProtKB)/Swiss-Prot database (Bairoch and Apweiler, 2000; Boutet et al., 2007). The dataset was parsed and classified into TFs and non-TFs using the Gene Ontology (GO) annotation. A protein sequence entry was annotated as a TF if it met the following criteria: a) the entry has a GO annotation for TF activity, or b) the entry has both a DNA-binding-related GO annotation and a transcription regulation-related GO annotation. The complete table for GO terms used to classify the TFs and non-TFs is provided in Supplementary Table S1. Here, we obtained 21,802 TF sequences and 539,374 non-TF sequences. We have developed a generalized method to predict the transcription factor. Therefore, we included transcription factor sequences from a diverse array of organisms. Nearly 9% of the transcription factor sequences in our dataset belong to *Homo sapiens*, about 8% are derived from *Arabidopsis thaliana*, approximately 6% come from *Mus musculus*, and around 2% are from *Rattus norvegicus*. The remaining sequences encompass a variety of other organisms, ensuring a broad and comprehensive dataset that supports the generalization capabilities of TransFacPred. This diverse inclusion aims to facilitate accurate transcription factor prediction across different species, paving the way for future developments that may include organism-specific methods to further refine and enhance prediction accuracy. We removed redundant sequences and sequences with non-natural amino acids from the TF and non-TF datasets. For the positive dataset, we obtained 19,406 unique TF sequences out of 21,802 sequences. For the negative dataset, we obtained 523,560 non-TF sequences from 539,374 entries. The final dataset comprises 19,406 TFs (positive) and 523,560 non-TFs (negative) protein sequences. Then, we followed the standards used in previous studies (Dhall et al., 2021; Dhall et al., 2022) and split the whole dataset into an 80% training dataset comprising 434,373 sequences (15,525 TFs and 418,848 non-TFs) and a 20% independent dataset containing 108,594 sequences (3,882 TFs and 104,712 non-TFs). As of June 2024, the March 2024 release of Swiss-Prot contains a total of 571,609 proteins, of which 25,052 have been designated as transcription factors based on the above-mentioned criteria. After processing these transcription factor protein sequences, we had a total of 21,125 sequences after removing the redundant sequences and sequences with non-natural amino acids. Among these, 1719 sequences were newly identified and were not available in the September 2019 release. These new sequences, along with additional relevant information, are detailed in Supplementary Table S2.

### 2.2 Feature generation

#### 2.2.1 Composition-based features

Pfeature (Pande et al., 2023) was used in this study to compute the amino acid composition- (AAC) and dipeptide composition (DPC)-based features of positive and negative datasets. In the case of AAC, a feature vector of length 20 was generated (using Eq. 1), which represents the composition of 20 amino acids in the sequence. Dipeptide composition is used to encapsulate the global information about each sequence, which gives a fixed vector of length 400 (20 × 20) using Eq. 2.

$$AAC_{i}=\frac{R_{i}}{L}$$ (1)

where $AAC_{i}$ is the AAC of residue type i ; $R_{i}$ and L are the number of residues of type i and the length of the sequence, respectively.

$$DPC_{i}=\frac{D_{i}^{j}}{L-j}$$ (2)

where $DPC_{i}$ is the fraction or composition of a dipeptide of type i for j th order. $D_{i}^{j}$ and L are the number of dipeptides of type i and the length of a protein sequence, respectively.

#### 2.2.2 One-hot encoding (OHE)

We implemented one-hot encoding approach for feature generation using TF and non-TF sequences. It is a representation of categorical variables as binary vectors. First, it requires that the categorical values be mapped to integer values. Then, each integer value is represented as a binary vector that is all zero values except the index of the integer, which is marked with a 1. In OHE, each amino acid is represented by the vector size of length 21; for instance, A is described as 1,0,0,0,0,0,0,0,0,0,0,0,0,0,0,0,0,0,0,0,0; which consists of 20 natural amino acids and one dummy variable, whereas X is represented as 0,0,0,0,0,0,0,0,0,0,0,0,0,0,0,0,0,0,0,0,0.

### 2.3 Model development

We implemented a number of classifiers to develop prediction models to predict the transcription factors using sequence information. Here, we used Scikit-learn-based traditional machine learning algorithms such as decision tree (DT), eXtreme gradient boosting (XGB), random forest (RF), Gaussian naïve Bayes (GNB), K-nearest neighbor (KNN), extra tree (ET), logistic regression (LR), and support vector classifier (SVC). We implemented a variety of classifiers based on different algorithms, such as DT, RF, and ET, which are tree-based approaches. DT is a non-parametric supervised learning method. It works by splitting the data into subsets based on the most significant feature at each node, leading to a tree-like model of decisions. RF is an ensemble method that constructs multiple decision trees during training. It outputs the class, which is the mode of the classes of the individual trees, improving predictive accuracy and controlling overfitting. ET is similar to RF but differs in the way splits are chosen. ET selects splits randomly, reducing variance and improving the model’s robustness. XGB is a boosting-based approach; it is an advanced implementation of gradient boosting. It builds trees sequentially, with each tree correcting errors from the previous trees, leading to high predictive performance and robustness against overfitting. GNB is a Bayesian-based approach that is based on Bayes’ theorem with the assumption of feature independence. It models the distribution of the data using Gaussian distributions. KNN is an instance-based learning method that classifies a sample based on the majority label among its closest neighbors in the feature space. It is simple and effective but can be computationally intensive. LR models the probability of a binary outcome using a logistic function. It is a linear model used for binary classification, where the output is interpreted as the probability of a particular class. SVC constructs hyperplanes in a high-dimensional space to separate different classes. It optimizes the margin between the classes, which helps improve classification accuracy and generalization.

We employed a hyperparameter tuning technique using the grid search approach available in Python’s Scikit-learn library to identify the optimal parameters for each classifier. This method exhaustively searches over a specified parameter grid to determine the best combination of parameters that yields the highest performance for each model. The most effective parameters and their corresponding values, as determined by grid search, are documented in Supplementary Table S3. This table provides a comprehensive overview of the tuned parameters for each classifier, ensuring reproducibility and transparency of the results.

### 2.4 Five-fold cross-validation

To avoid the curse of biases and overfitting of models, we performed five-fold cross-validation on the training dataset (Patiyal et al., 2020; Dhall et al., 2021; Patiyal et al., 2022). In this approach, the training dataset is stratified into five sets, where the model is trained on four sets and tested on the remaining one. The same process is repeated five times in such a way that each set acts as a testing dataset. The final performance is the average of performances resulting from each iteration.

### 2.5 Similarity search approach

We also implemented similarity search using BLAST (McGinnis and Madden, 2004), a widely used tool to annotate the sequences. We used it to classify the sequences as transcription factors or non-transcription factors based on their similarity. The BLASTP suite of NCBI-BLAST + version 2.2.29 was used to perform the similarity search. The training dataset was used to create the custom database, and the makeblastdb application of NCBI-BLAST+ was used for the same. Sequences in the independent dataset were hit against the custom database to assign the class as a transcription factor or non-transcription factor based on their similarity with the sequences in the database. We considered the top hit of BLAST to assign the classes, such that if the top hit of the BLAST is against the transcription factor sequence of the database, then the query protein is assigned as a transcription factor; otherwise, it was labeled as a non-transcription factor. We ran the BLAST at different e-value cut-offs varying from 1e − 6 to 1e + 3 in order to find the optimal value to classify the transcription factors.

### 2.6 Performance evaluation

We used various performance evaluation parameters such as accuracy, sensitivity, specificity, F1-score, area under the receiver operating characteristics curve (AUC), and Matthews correlation coefficient (MCC). Sensitivity (see Eq. (3)), specificity (see Eq. (4)), accuracy (see Eq. 5), F1-score (see Eq. (6)), and MCC (see Eq. (7)) are threshold-dependent parameters. In contrast, AUC is a threshold-independent parameter. The various performance evaluation parameter equations are provided below.

$$Sensitivity=\frac{TP}{TP+FN}*\;100$$ (3)

$$Specificity=\frac{TN}{TN+FP}\;*100$$ (4)

$$Accuracy=\frac{TP+TN}{TP+FP+TN+FN}*100$$ (5)

$$F1-score=\frac{2TP}{FP+FN}$$ (6)

$$MCC=\frac{\left(TP*TN\right)\;-\;\left(FP*FN\right)}{\sqrt{\left(TP+FP\right)\left(TP+FN\right)\left(TN+FP\right)\left(TN+FN\right)}}$$ (7)

where FP is False Positive, FN is False Negative, TP is True Positive, and TN is True Negative.

## 3 Results

### 3.1 Compositional analysis

**FIGURE 1:**
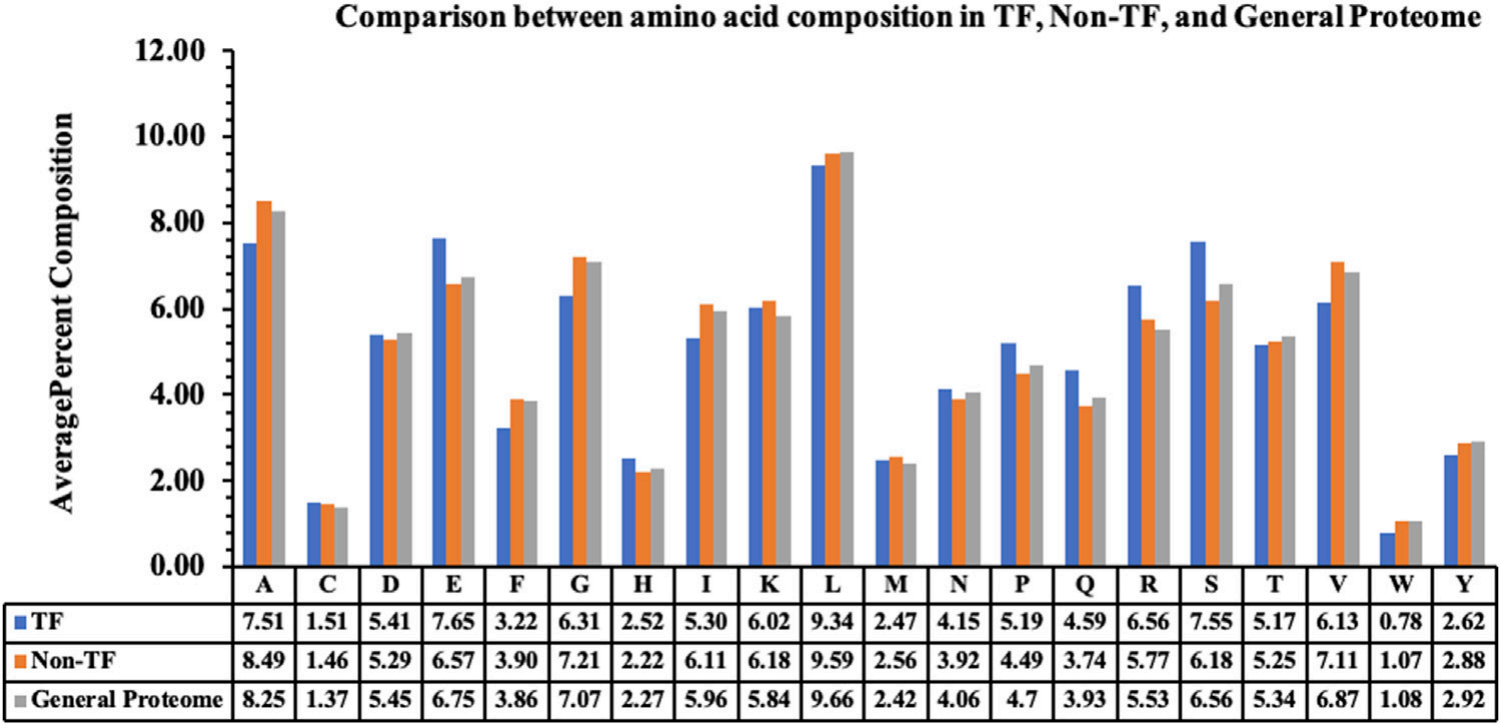
Average percent composition of amino acid residues in TFs, Non-TFs, and the general proteome.

We performed the amino acid-based compositional analysis for the TF, non-TF, and general proteome classes to compare the abundance of the residues in these classes. Figure 1 represents the average percent composition of each residue in proteins belonging to the TF and non-TF classes. It compares the same with the average percent composition of general proteome derived from the Swiss-Prot database. As exhibited by the bar plot, transcription factors are rich in E, P, Q, R, and S residues compared to the non-transcription factors, whereas residues A, G, I, and V are abundant in non-transcription factor proteins.

### 3.2 Performance on alignment-based method

To classify the transcription factors using an alignment-based method, we performed the similarity search using BLAST by varying the e-value from 1.00E−06 to 1.00E+03. In this approach, we created the database using the sequences in the training dataset, hit the query proteins in the independent dataset against it, and considered the top hit to assign the class to each query protein. The performance at each value is reported in Table 1. As shown in Table 1, BLAST achieved a good performance for predicting the transcription factors but could not cover the entire dataset. Moreover, as the e-value increases, the probability of a correct prediction decreases. Hence, BLAST alone is not sufficient for predicting the transcription factors.

**TABLE 1:** Performance on alignment-based approach at different e-values.

E-Value	No hits [positive]	Probability of correct prediction
1.00E−06	68	95.44
1.00E−05	57	95.40
1.00E−04	44	95.28
1.00E−03	39	95.29
1.00E−02	32	95.30
1.00E−01	29	95.28
1.00E+00	22	95.26

### 3.3 Performance on alignment-free methods

We implemented eight traditional machine learning classifiers, such as DT, RF, LR, XGB, GNB, KNN, ET, and SVC, using various features like AAC, DPC, and AAC + DPC as the input feature to classify the protein sequences into TFs and non-TFs. We trained the model on the 80% training dataset and evaluated its performance on the remaining 20% independent dataset. First, we developed various prediction models using AAC, and the performance of each classifier is reported in Table 2. As shown by Table 2, the ET-based model outperforms the other models with an AUC of 0.97 on the training and independent datasets with balanced sensitivity and specificity.

**TABLE 2:** Performance of various classifiers using AAC as the input feature.

Classifier	Training dataset							Independent dataset						
	Sens	Spec	Acc	AUC	F1	K	MCC	Sens	Spec	Acc	AUC	F1	K	MCC
DT	52.435	98.192	96.557	0.753	0.523	0.505	0.505	52.486	98.273	96.637	0.754	0.529	0.511	0.511
RF	91.028	89.057	89.127	0.964	0.708	0.698	0.701	91.961	89.195	89.294	0.968	0.721	0.711	0.713
LR	73.895	74.756	74.725	0.814	0.214	0.168	0.215	74.130	75.001	74.970	0.813	0.213	0.169	0.215
XGB	85.592	86.791	86.748	0.940	0.582	0.568	0.574	86.988	86.966	86.967	0.946	0.589	0.575	0.583
KNN	84.684	94.997	94.628	0.913	0.671	0.661	0.669	85.803	95.011	94.682	0.919	0.674	0.663	0.672
GNB	67.835	71.707	71.569	0.772	0.235	0.202	0.206	67.070	72.002	71.825	0.767	0.235	0.201	0.206
ET	90.461	90.866	90.852	0.967	0.733	0.724	0.729	91.033	90.949	90.952	0.968	0.745	0.736	0.740
SVC	80.246	80.812	80.791	0.891	0.488	0.469	0.470	81.474	81.186	81.196	0.897	0.489	0.469	0.470

^a^ AAC: Amino acid composition; DT: Decision tree; RF: Random forest; LR: Logistic regression; XGB: eXtreme gradient boosting; KNN: K-nearest neighbor; GNB: Gaussian naïve Bayes; ET: Extra trees; SVC: Support vector classifier; Sens: Sensitivity; Spec: Specificity; ACC: Accuracy; AUC: Area under the receiver operating characteristics curve; K: kappa; MCC: Matthews correlation coefficient.

Similarly, various machine learning models were developed to classify TFs using DPC as the input feature. Table 3 represents the performance of models based on each classifier, and the model based on the XGB classifier performed best among the other classifiers with an AUC of 0.96 on the training and validation dataset.

**TABLE 3:** Performance of various classifiers using DPC as the input feature.

Classifier	Training dataset							Independent dataset						
	Sens	Spec	Acc	AUC	F1	K	MCC	Sens	Spec	Acc	AUC	F1	K	MCC
DT	52.544	98.180	96.549	0.754	0.522	0.505	0.505	52.409	98.193	96.557	0.753	0.522	0.504	0.504
RF	90.648	89.220	89.271	0.964	0.720	0.710	0.715	90.518	89.444	89.482	0.964	0.728	0.719	0.726
LR	80.343	80.711	80.698	0.876	0.301	0.265	0.303	80.752	80.779	80.778	0.878	0.308	0.272	0.309
XGB	90.113	90.305	90.298	0.965	0.720	0.710	0.715	90.054	90.505	90.488	0.966	0.720	0.711	0.716
KNN	84.117	96.321	95.885	0.913	0.714	0.703	0.704	83.767	96.225	95.780	0.912	0.719	0.709	0.709
GNB	75.866	49.497	50.439	0.694	0.166	0.122	0.135	76.269	49.668	50.619	0.696	0.165	0.122	0.133
ET	90.938	88.708	88.788	0.965	0.757	0.749	0.752	90.673	88.889	88.952	0.964	0.756	0.747	0.753
SVC	88.864	92.169	92.051	0.960	0.781	0.774	0.778	89.307	92.171	92.069	0.964	0.787	0.779	0.782

^a^ DPC: Dipeptide composition; DT: Decision tree; RF: Random forest; LR: Logistic regression; XGB: eXtreme gradient boosting; KNN: K-nearest neighbor; GNB: Gaussian naïve Bayes; ET: Extra trees; SVC: Support vector classifier; Sens: Sensitivity; Spec: Specificity; ACC: Accuracy; AUC: Area under the receiver operating characteristics curve; K: Kappa; MCC: Matthews correlation coefficient.

In the next step, we combined the AAC and DPC features, which resulted in a vector of size 420 for each protein, and developed prediction models. We used eight different classifiers, and their performance is reported in Table 4. Similar to the performance on individual features, the XGB-based model performed best among all the other classifiers with an AUC of 0.97 on the training and independent datasets.

**TABLE 4:** Performance of various classifiers using a combination of AAC and DPC as the input feature.

Classifier	Training dataset							Independent dataset						
	Sens	Spec	Acc	AUC	F1	K	MCC	Sens	Spec	Acc	AUC	F1	K	MCC
DT	54.412	98.270	96.703	0.763	0.543	0.526	0.526	53.491	98.287	96.686	0.759	0.537	0.519	0.519
RF	91.885	89.655	89.735	0.969	0.729	0.720	0.723	91.832	89.731	89.806	0.969	0.738	0.729	0.732
LR	80.845	80.190	80.214	0.875	0.295	0.259	0.298	80.881	80.282	80.304	0.878	0.303	0.268	0.303
XGB	90.815	90.592	90.600	0.969	0.735	0.726	0.731	91.007	90.675	90.687	0.970	0.736	0.727	0.731
KNN	85.611	96.317	95.934	0.921	0.719	0.708	0.712	85.442	96.389	95.998	0.920	0.722	0.711	0.711
GNB	76.188	50.433	51.353	0.704	0.180	0.135	0.155	76.424	50.612	51.534	0.706	0.182	0.137	0.156
ET	91.814	88.980	89.082	0.968	0.758	0.750	0.754	91.497	89.178	89.261	0.966	0.759	0.751	0.754
SVC	86.507	84.927	84.984	0.935	0.645	0.633	0.637	86.756	85.212	85.267	0.939	0.650	0.638	0.639

^a^ AAC: Amino acid composition; DPC: Dipeptide composition; DT: Decision tree; RF: Random forest; LR: Logistic regression; XGB: eXtreme gradient boosting; KNN: K-nearest neighbor; GNB: Gaussian naïve Bayes; ET: Extra trees; SVC: Support vector classifier; Sens: Sensitivity; Spec: Specificity; ACC: Accuracy; AUC: Area under the receiver operating characteristics curve; K: Kappa; MCC: Matthews correlation coefficient.

### 3.4 Performance of deep learning models

We also developed deep learning technique-based prediction models to classify the TFs using different features such as AAC, DPC, AAC + DPC, and one-hot encoding (OHE). Table 5 exhibits the performance of the different models on the validation datasets using different features. As shown in Table 5, the CNN-based model with one-hot encoding as the input feature performed best with an AUC of 0.95 on the independent dataset.

**TABLE 5:** Performance of convolutional neural network-based model using various features on the independent dataset.

Feature	Sensitivity	Specificity	Accuracy	AUC	F1	K	MCC
AAC	8.00	99.00	96.30	0.54	0.14	0.14	0.24
DPC	53.22	99.73	97.92	0.76	0.67	0.66	0.68
AAC + DPC	59.34	99.49	97.93	0.79	0.69	0.68	0.69
OHE	91.27	98.61	98.32	0.95	0.81	0.81	0.81

^a^ AAC: Amino acid composition; DPC: Dipeptide composition; OHE: One-hot encodings; AUC: Area under the receiver operating characteristics curve; K: Kappa; MCC: Matthews correlation coefficient.

### 3.5 Performance of hybrid (alignment-based + alignment-free) model

We also developed a hybrid model for classifying transcription factors by combining alignment-free and alignment-based approaches. The alignment-free component employs machine learning classifiers, while the alignment-based component utilizes similarity search with BLAST, resulting in a more accurate and comprehensive prediction method. In the hybrid approach, we combined the outputs from the ET-based model developed using amino acid composition and BLAST search to make the final prediction. Table 6 exhibits the performance of the hybrid model at different e-values on the independent dataset. As shown in Table 6, at each e-value, the AUC achieved was 0.99, with balanced sensitivity and specificity; in terms of accuracy, an e-value of 1.00E + 02 attained the maximum value of 97.013%. This model has been incorporated into the backend of the server TransFacPred to predict if the submitted protein is a TF or a non-TF.

**TABLE 6:** Performance of hybrid method (AAC + BLAST) on the independent dataset.

E-value	Sensitivity	Specificity	Accuracy	AUC	F1	K	MCC
1.00E−06	95.88	95.41	95.42	0.99	0.94	0.93	0.93
1.00E−05	95.83	95.56	95.57	0.99	0.94	0.93	0.93
1.00E−04	95.70	95.76	95.76	0.99	0.94	0.94	0.94
1.00E−03	95.70	95.93	95.92	0.99	0.94	0.94	0.94
1.00E−02	96.03	96.03	96.03	0.99	0.94	0.94	0.94
1.00E−01	96.16	96.18	96.18	0.99	0.94	0.94	0.94
1.00E+00	96.34	96.41	96.41	0.99	0.94	0.94	0.94
1.00E+01	96.96	96.76	96.77	0.99	0.93	0.93	0.93
1.00E+02	97.06	97.01	97.01	0.99	0.93	0.92	0.92
2.00E+02	97.06	97.15	97.15	0.99	0.93	0.92	0.92
1.00E+03	97.06	97.24	97.24	0.99	0.93	0.92	0.92

^a^ AAC: Amino acid composition; AUC: Area under the receiver operating characteristics curve; MCC: Matthews correlation coefficient.

### 3.6 Comparison with existing methods

To understand the advantages or disadvantages of the newly proposed method, it is crucial to compare it with the existing methods. Hence, we compared the performance of our model with the published methods such as DeepTFactor, TFpredict, and P2TF (Ortet, et al., 2012; Eichner et al., 2013; Kim et al., 2021). We evaluated our and existing models on the independent dataset, and as signified in Table 7, our model performed better in terms of each evaluation parameter.

**TABLE 7:** Comparison of the performance of our best-performing model with existing tools on the independent dataset.

Parameters	TransFacPred	DeepTFactor	TFpredict	P2TF
Sensitivity	97.06	95.93	93.41	92.84
Specificity	97.01	95.78	92.85	86.16
Accuracy	97.01	95.79	92.87	86.40
AUC	0.99	0.97	0.94	0.88
F1	0.93	0.85	0.48	0.33
K	0.92	0.84	0.48	0.32
MCC	0.92	0.85	0.53	0.40

^a^ AUC: Area under the receiver operating characteristics curve; K: Kappa; MCC: Matthews correlation coefficient.

Additionally, we compared the processing times of the recently published DeepTFactor with our proposed method, TransFacPred, using both standalone machine learning and a hybrid model. By testing various numbers of sequences simultaneously, we found that DeepTFactor takes longer as the number of sequences increases, as shown in Table 8. We implemented the AAC-based machine learning model and a hybrid model and compared the performance. The ML-based model took less time than DeepTFactor with an equivalent AUC, whereas the hybrid model performed best but took more time to provide the output.

**TABLE 8:** Comparison between the processing time of DeepTFactor and TransFacPred.

Number of sequences	Method	Time (in seconds)		
		Real	User	System
50	DeepTFactor	13.285	3.882	1.188
	TransFacPred [ML]	7.666	1.551	0.998
	TransFacPred [Hybrid]	24.111	22.079	1.254
1,000	DeepTFactor	55.201	51.37	3.954
	TransFacPred [ML]	37.208	2.649	1.157
	TransFacPred [Hybrid]	436.071	429.062	3.157
108,594	DeepTFactor	6014.113	5629.047	375.138
	TransFacPred [ML]	134.387	130.191	1.945
	TransFacPred [Hybrid]	47,932.78	47,583.942	304.83

^a^ ML: Machine learning.

### 3.7 Web server implementation

**FIGURE 2:**
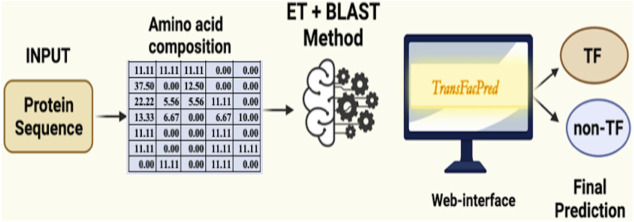
Graphical representation of the the TransFacPred web server using a hybrid model.

**FIGURE 3:**
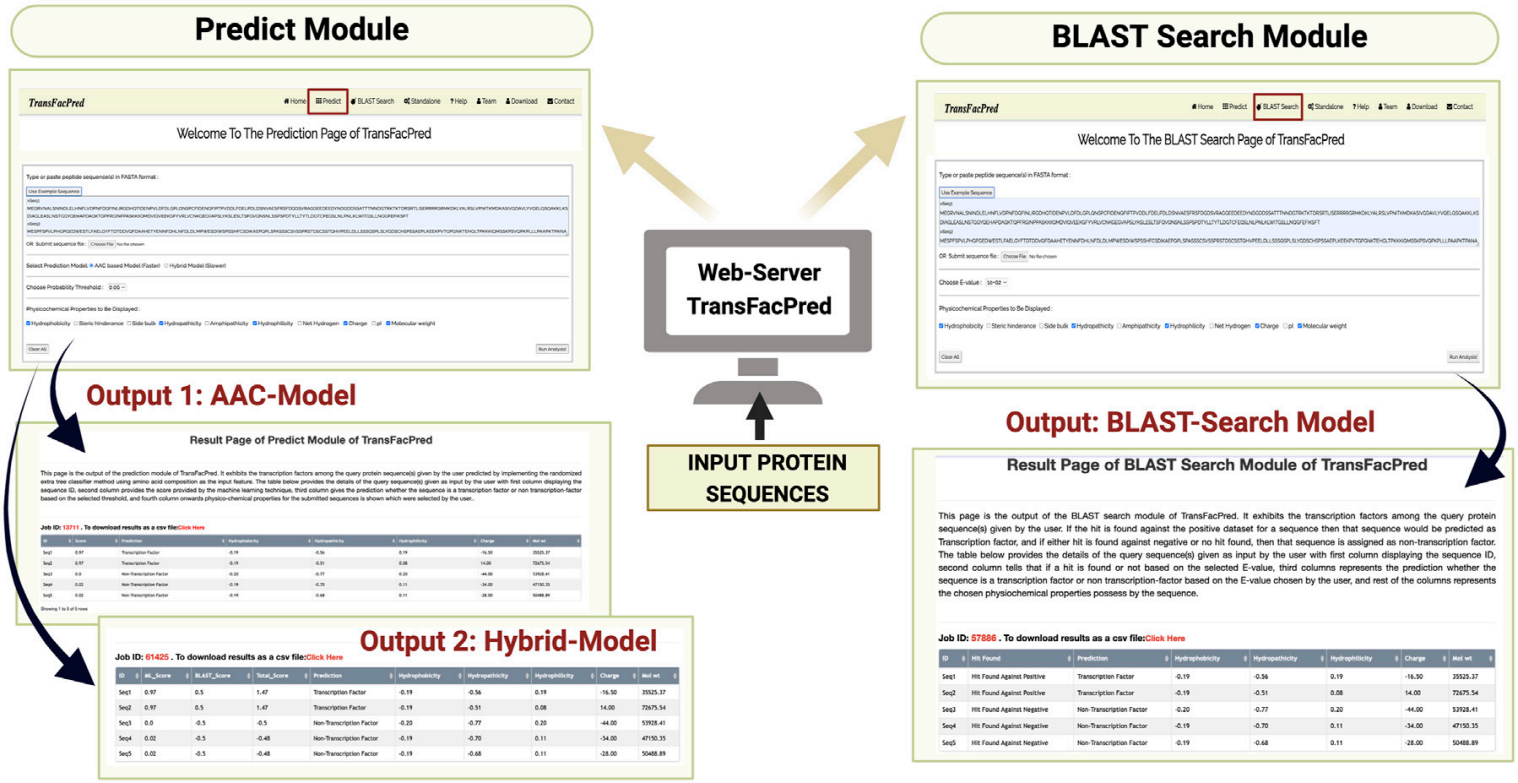
Usage of the Predict and BLAST Search modules of TransFacPred.

We developed an easy-to-use web server, TransFacPred, and a standalone package. Our web server has two major modules: Predict and BLAST Search. The predictive module allows the users to predict TFs using an alignment-free method or a hybrid method (see Figure 2). The BLAST search module allows users to perform a BLAST search against the database of TFs and non-TFs used in this study. The comprehensive utility of the BLAST Search module and predict module using the AAC- and hybrid-based model is shown in Figure 3. In addition to the web server, we developed a standalone package in Python. This package is suitable for scanning TFs at the genome scale, where it can be run on a local machine.

## 4 Discussion

TFs initiate the transcription process and hence play a major role in deciding the fate of a cell or cellular process (Rhee et al., 2017; Islam et al., 2021). Identification of novel or unknown TFs using experimental-based techniques such as RNA sequencing (RNA-seq) and Chromatin immunoprecipitation sequencing (ChIP-seq) experiment is a tiring and expensive task (Muhammad et al., 2019). Previously, a number of methods have been developed for the prediction of TFs (Zheng et al., 2008; Eichner et al., 2013; Kim et al., 2021). To assist the researchers working in this field, we made a systematic attempt to develop a highly accurate method capable of classifying TFs using the primary sequence information. Based on GO terms, sequences were assigned as either TFs or non-TFs. At first, there was a total of 561,176 sequences, of which 21,802 were assigned as TFs and 539,374 were designated as non-TFs; after preprocessing the datasets, the final dataset was comprised of 19,406 TFs and 523,560 non-TFs. These sequences are from diverse organisms, which signifies the diversity in the proposed model. Of the TF sequences, approximately 9% are from *H. sapiens*, 8% from *A. thaliana*, 6% from *M. musculus*, 2% from *R. norvegicus*, and the rest belong to other organisms.

In this study, we employed an imbalanced dataset to train and evaluate the models, where the number of TFs was significantly higher than the number of TF sequences. It is crucial to understand that the use of either balanced datasets or imbalanced/realistic datasets is pertinent not only to this study but to all similar studies. Previous research has thoroughly discussed the importance of both balanced and realistic datasets (Agarwal et al., 2011; Agrawal et al., 2020; Patiyal et al., 2020). Notably, a balanced dataset is essential for training, testing, and evaluating any supervised machine learning technique as it ensures equal preference to all classes. Many data scientists favor using balanced datasets because they facilitate the training and evaluation process through straightforward metrics such as accuracy. However, in real-world scenarios, classes are often imbalanced. For instance, there are typically far more non-transcription factors than transcription factors. A model trained on a balanced dataset might try to predict an equal number of transcription factors and non-transcription factors in a given protein set, which does not represent the real situation accurately. Biologists and other domain experts often prefer to train machine learning models on realistic datasets that reflect the inherent imbalance found in real-world data. However, training such models presents challenges because machine learning techniques tend to favor classes with more samples. Furthermore, simple metrics like accuracy may not be sufficient to evaluate such models adequately. To address this issue, we evaluated the models in this study using metrics that penalize over-prediction and account for class imbalances, such as the MCC. This approach ensures a more accurate and fair evaluation of the models’ performance, highlighting the importance of considering dataset composition in machine learning studies. Although the proposed model was developed using the sequences from an array of organisms, which led to the development of the general model, it is important to recognize that organism-specific methods may provide more precision than general methods. Initially, most methods were developed for a wide range of organisms, but they were later replaced by organism-specific methods due to their better accuracy. For example, in the field of subcellular localization, methods were initially developed for the subcellular localization of eukaryotic proteins, such as ESLpred (Garg and Raghava, 2008). Later, organism-specific methods were developed, such as for human proteins (Zhang et al., 2022) and RSLpred for rice proteins (Kaundal and Raghava, 2009).

**FIGURE 4:**
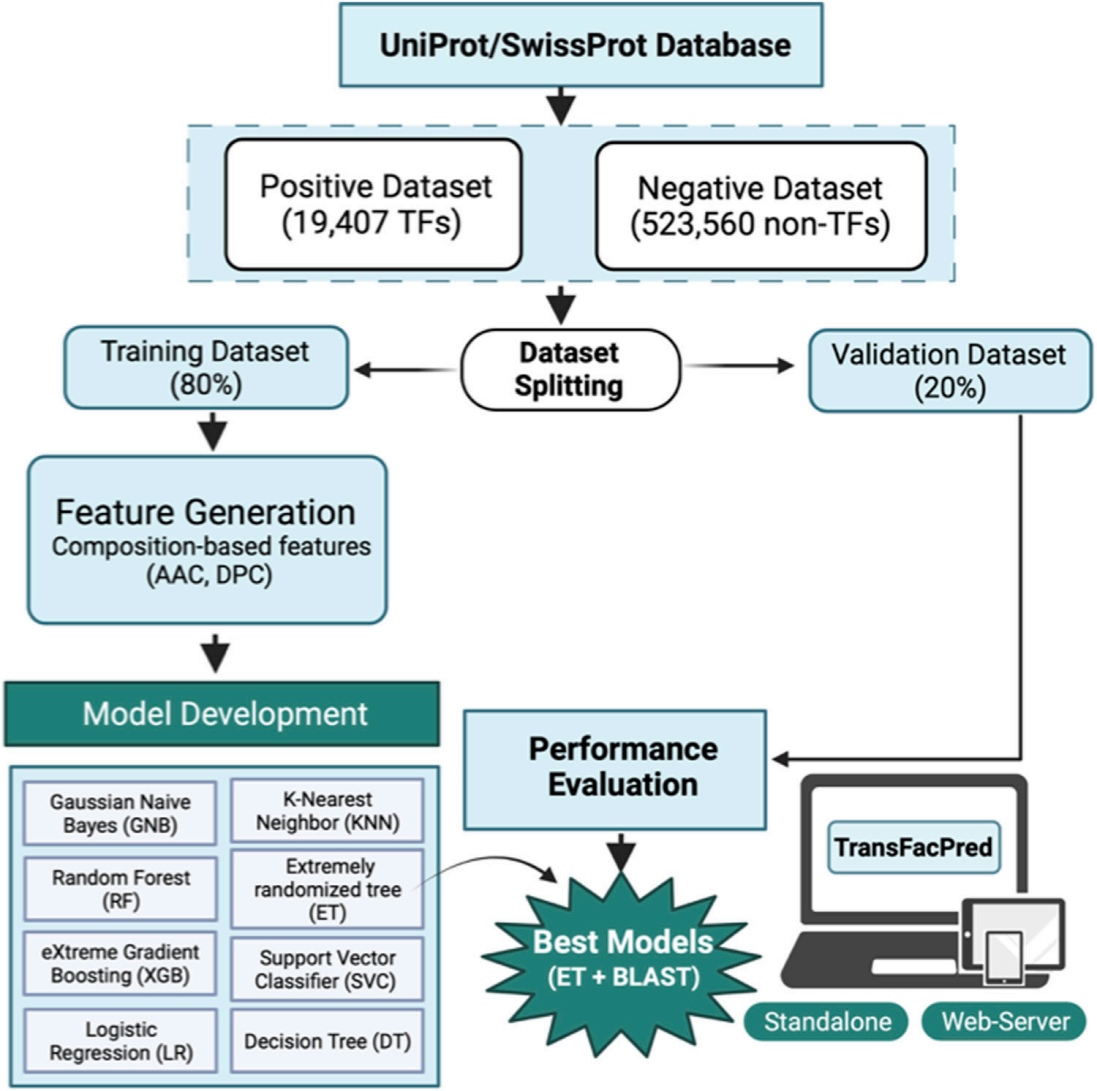
Complete workflow for TransFacPred.

The preliminary composition analysis on this dataset showed that the TFs are rich in E, P, Q, R, and S amino acids. Further, sequence-based features were computed using Pfeature software, and various machine learning techniques were implemented to exploit their capabilities to classify the sequences as either TFs or non-TFs. Our models were trained on 80% of the dataset using different sets of features and validated on the remaining previously unseen 20% of the dataset. JWe obtained an AUC of 0.96 on the training and on an independent dataset using amino acid composition-based features. Of all the models, the hybrid model, which is the combination of the ET-based model developed on amino acid composition and BLAST search, performed best with an AUC of 0.99 on the independent dataset with balanced sensitivity and specificity. We also compared our method with the existing methods such as DeepTFactor, TFpredict, and P2TF to predict the transcription factors using sequence information. We trained our models on the training dataset and evaluated the performance of the TransFacPred and existing approaches on the independent dataset. We demonstrated that the proposed model of TransFacPred outperformed the existing approaches to classify the TFs in terms of AUC and other parameters. We anticipate that this research will aid researchers working in genomics and proteomics. Figure 4 represents the complete flow of this study.

## 5 Potential applications of TransFacPred

TransFacPred has applications in many different areas of biological study. Accurate identification of transcription factors enables researchers to focus on functional analysis and regulatory mechanisms, thereby deepening the understanding of cellular processes and gene expression regulation (Davidson and Erwin, 2006). For instance, transcription factors play crucial roles in controlling developmental processes, responding to environmental stimuli, and regulating cellular differentiation (Lee and Young, 2013). By integrating TransFacPred into genomic studies, researchers can expedite the identification of transcription factors, facilitating a more efficient analysis of large datasets and complex biological systems (Vaquerizas et al., 2009). In practical genomic data analysis, TransFacPred can annotate newly sequenced genomes, assisting in the rapid identification of transcription factors (Wasserman and Sandelin, 2004). This tool is particularly beneficial in comparative genomics, where researchers aim to elucidate evolutionary relationships and functional conservation of transcription factors across different species (Levine and Tjian, 2003). For instance, predicting transcription factors in novel genomes can reveal insights into regulatory networks and gene expression patterns across diverse organisms, contributing to our understanding of evolutionary biology and functional genomics (Wray et al., 2003). Furthermore, TransFacPred can be used in metagenomic studies to identify transcription factors in microbial communities, shedding light on the regulatory mechanisms underlying microbial diversity and ecosystem functions (Moran et al., 2013). In oncology, TransFacPred could be utilized to identify transcription factors involved in cancer development and progression. For example, studies have shown that transcription factors such as MYC and TP53 play critical roles in tumorigenesis (Vousden and Lane, 2007; Dang, 2012). By analyzing protein sequences from tumor samples, TransFacPred can help to select the key regulatory proteins that may serve as potential biomarkers or therapeutic targets, thereby aiding in the development of targeted cancer therapies.

TransFacPred can aid in agricultural studies by identifying transcription factors that regulate stress response and developmental pathways in plants. Transcription factors like DREB and WRKY have been associated with stress responses in crops, playing crucial roles in plant adaptation to abiotic stresses such as drought, salinity, and cold (Yamaguchi-Shinozaki and Shinozaki, 2006; Rushton et al., 2010). This information is valuable for engineering crops with enhanced resistance to environmental stresses, leading to improved yield and sustainability (Hirayama and Shinozaki, 2010). For example, overexpression of DREB1A in transgenic rice has been shown to enhance drought and cold tolerance, demonstrating the practical application of transcription factor research in crop improvement (Datta et al., 2012). Understanding the role of transcription factors in developmental processes is crucial for developmental biology studies. For instance, transcription factors such as SOX2 and OCT4 are key regulators of stem cell pluripotency and differentiation (Masui et al., 2007; Nichols and Smith, 2012). SOX2 and OCT4 form a core regulatory network that maintains the pluripotent state of embryonic stem cells and regulates their differentiation into various cell types (Masui et al., 2007). Disruptions in these transcription factors can lead to developmental disorders and diseases, highlighting their importance in developmental biology (Nichols and Smith, 2012). TransFacPred can assist in identifying key transcription factors involved in differentiation and morphogenesis, providing insights into developmental disorders and regenerative medicine (Slack, 1995).

## 6 Limitations of the study

While TransFacPred offers substantial benefits, it is essential to acknowledge its limitations. The accuracy of predictions may vary depending on the quality and diversity of input protein sequences. TransFacPred’s performance might be constrained by the availability of comprehensive training data, which could impact its ability to generalize across different organisms and conditions. Moreover, the predictive models used by TransFacPred might not fully capture the complex regulatory interactions and context-dependent activities of transcription factors, necessitating experimental validation to confirm the biological relevance of the predictions. It is also important to consider the potential biases introduced by the training data, which might affect TransFacPred’s applicability to novel or underrepresented species. Although TransFacPred can identify whether an input protein sequence is a transcription factor, it does not provide information about the binding site or affinity scores. Furthermore, while we have provided a generalized model to predict transcription factors by including sequences from various organisms, it is important to recognize that transcription factors in different organisms may have distinct properties and functions. Therefore, it may be possible to develop organism-specific methods to predict transcription factors more accurately.

## Data Availability

Publicly available datasets were analyzed in this study. This data can be found here: https://webs.iiitd.edu.in/raghava/transfacpred/dataset.php.
